# Case Report: Unique case of lens replacement in high hyperopia with trifocal lens and piggyback light-adjustable lens implantation

**DOI:** 10.3389/fopht.2025.1641352

**Published:** 2025-12-10

**Authors:** Amirmohammad Shafiee, Arsalan A. Ali, Anirudh Gadicherla, Taj Nasser

**Affiliations:** 1 Medical Students, Anne Burnett Marion School of Medicine at Texas Christian University, Fort Worth, TX, United States; 2 Department of Ophthalmology, Loyola University Chicago Stritch School of Medicine, Maywood, IL, United States; 3 Parkhurst NuVision, San Antonio, TX, United States; 4 Tylock George Eye Care, Irving, TX, United States

**Keywords:** case report, light adjustable lens, piggyback lens, hyperopia, refractive surgery

## Abstract

Managing high hyperopia is notably challenging due to the unpredictability of refractive outcomes. Moreover, the technical difficulties of surgery in patients with smaller eyes, compounded by the limitations in available intraocular lens (IOL) powers in the United States, necessitate innovative approaches to achieve emmetropia. This case report discusses a novel approach using a light-adjustable lens (LAL) with a trifocal IOL to address these complexities and achieve spectacle independence. A 36-year-old man, who works as a military aircraft inspector and has high hyperopia and a history of left eye (OS) strabismic amblyopia, sought freedom from spectacles due to occupational demands. Pre-operative assessment showed severe uncorrected visual acuity and short axial lengths. Refractive lens exchange was performed bilaterally, utilizing a trifocal IOL combined with a light-adjustable lens OD and a high-powered monofocal lens OS. Post-operatively, significant improvement in visual acuity was observed, with heightened patient satisfaction. This case demonstrates the effectiveness of integrating a light-adjustable lens with a trifocal IOL to address high hyperopia in a less invasive manner and foster spectacle independence with patient satisfaction. Our approach offers promising implications for refractive surgery, highlighting the potential for tailored solutions in complex cases.

## Introduction

High hyperopia, which is defined as a refractive error exceeding +4.00 diopters (D), poses unique challenges in the field of refractive surgery. Among individuals aged 20 and older, the prevalence rates of hyperopia, myopia, and astigmatism are 3.6%, 33.1%, and 36.2%, respectively (1). This disparity is echoed in literature, where there are relatively few studies focusing on refractive lens exchange as a corrective measure for hyperopia compared to myopia (2). Moreover, conducting refractive surgery is technically difficult to execute on patients with smaller eyes and can also lead to a range of complications, including uveal effusion, aqueous misdirection, choroidal hemorrhage, and cystoid macular edema (3). Addressing high hyperopia is further complicated by challenges related to intraocular lens (IOL) calculations and the current limitations on available IOL powers in the United States (4). These issues may necessitate exploring alternative strategies to achieve emmetropia. For cases of severe hyperopia, where there is often a significant residual refractive error, the use of secondary IOLs or “piggyback” lenses can be considered to attain the desired refractive outcome (5). However, this approach comes with potential complications, such as interlenticular opacification or iris pigment dispersion. We present the first reported case of utilizing a light-adjustable lens (RxSight, RxLAL) in conjunction with a trifocal IOL on a 36-year-old man with a history of strabismic amblyopia in the left eye (OS) and bilateral high hyperopia to attain full refractive correction and spectacle independence.

## Case description

A 36-year-old man, employed as a military aircraft inspector, presented with a clinical history of strabismic amblyopia in the left eye (OS) and bilateral high hyperopia. The patient, desiring independence from spectacles, emphasized his daily work routine, which involved alternating near and distance vision tasks. Therefore, he was particularly interested in corrective solutions that would provide an extended range of vision.

**Figure 1:**
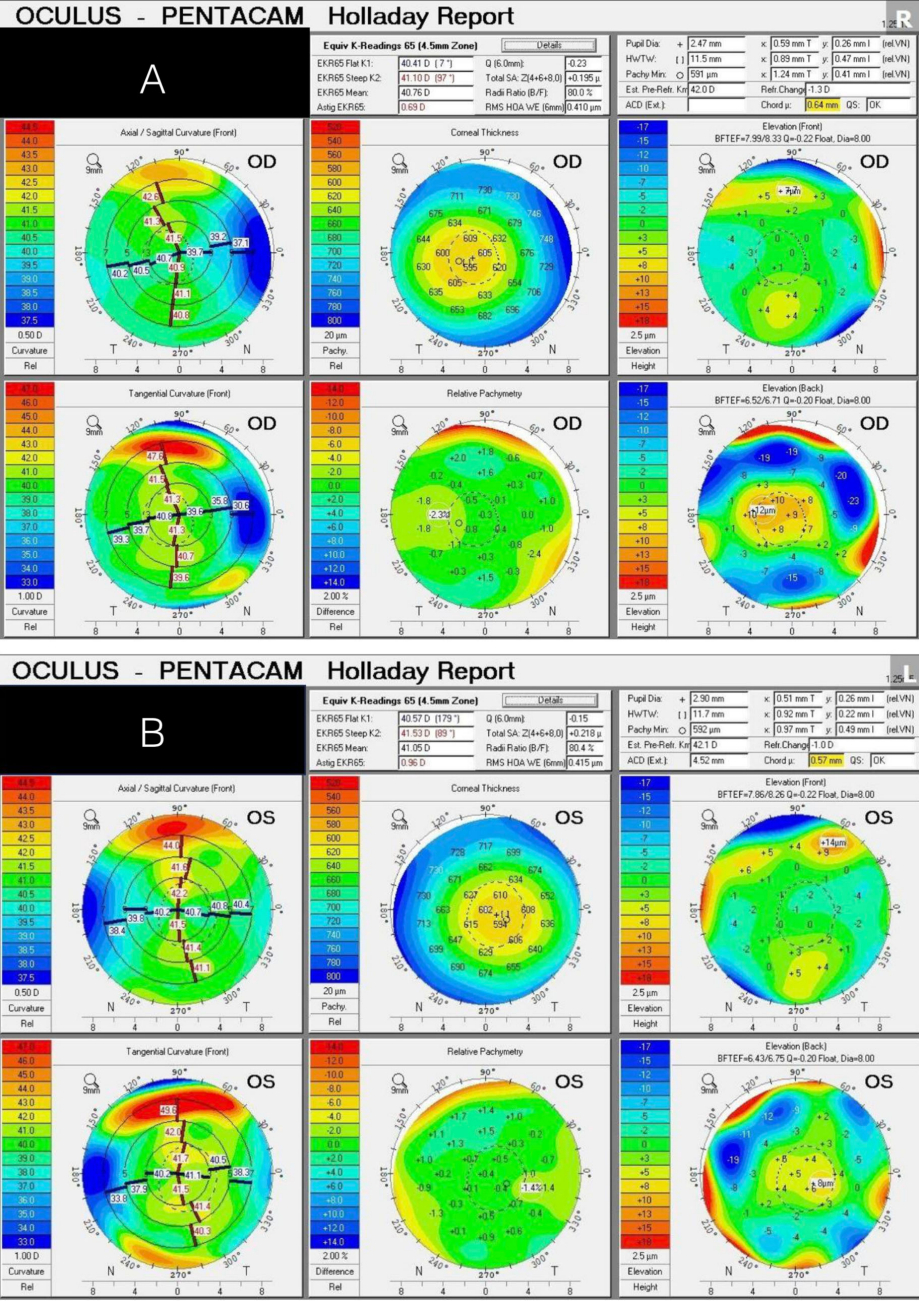
**(A, B)** Tomography with Holladay report showing expected large-angle kappa in the setting of high hyperopia and unremarkable higher-order aberrations.

A comprehensive external and slit lamp biomicroscopic examination revealed no abnormalities in either eye. Intraocular pressure and fundus examination were within normal limits. Extraocular movements were intact, and the eyes were orthophoric at primary. Cover/uncover and cross-cover tests were unremarkable. Pre-operatively, his uncorrected distance visual acuity (UDVA) was 20/400 in the right eye (OD) and counting fingers at 2 feet in OS. Manifest refraction (MRx) in OD was determined to be +10.00 sphere, resulting in a corrected distance visual acuity (CDVA) of 20/20. His MRx in OS was +11.50 −0.75 × 170, with a CDVA of 20/40. Optical biometry was obtained using Pentacam AXL (Oculus Optikgeräte GmbH, Wetzlar, Germany) and revealed an axial length of 20.1 mm and 19.7 mm in OD and OS, respectively. Pentacam measurements determined the internal anterior chamber depth (ACD) to be 2.05 mm in OD and 2.21 mm in OS. Tomography was obtained, as shown in **Figure 1**, and the OCT macula was unremarkable.

After an extensive discussion of the patient’s options and their potential limitations, including the loss of accommodation, reduction in image magnification, and reduction in contrast sensitivity, the patient elected to proceed with refractive lens exchange in both eyes. Refractive lens exchange was performed in the left eye, followed by the right eye, on the subsequent day. **Table 1** provides a sequential timeline of the patient's procedure.

**Table 1:** Timeline of patient surgery.

Date	Event
August 2023	Initial patient visit
August 2023	Piggyback LAL pre-op visit, visual acuity assessed
August 2023	OS LAL placement
August 2023	OD LAL placement
August 2023	1-day post-operative visual acuity was assessed
August 2023	1-week post-operative visual acuity was assessed
September 2023	1-month post-operative visual acuity was assessed

LAL, light-adjustable lens; OS, left eye.

**Figure 2:**
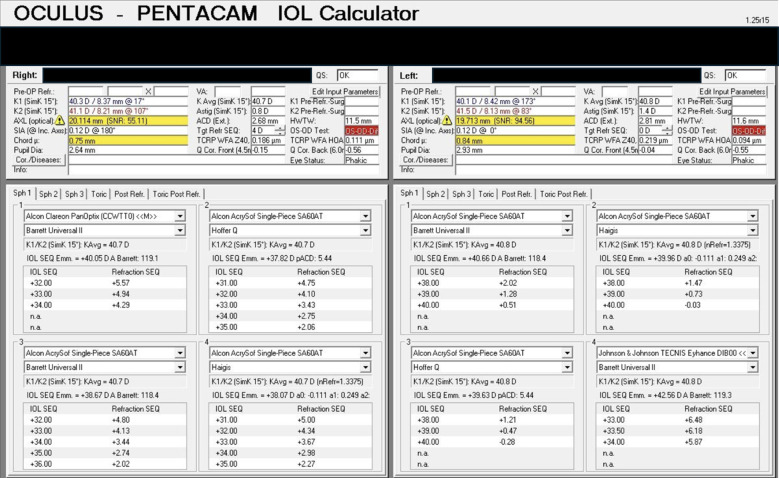
Pentacam AXL biometry measurements for both eyes with primary lens options selected. Note the significant variability in the predicted spherical equivalent.

In the left eye, a monofocal lens was selected due to the patient’s amblyopia in OS and the known reduction in contrast sensitivity associated with diffractive IOLs. Alcon SA60AT + 40.0 was chosen since it has the highest available power for a monofocal lens in the United States. Predicted spherical equivalent (SEQ) for several formulas used can be found in **Figure 2**. The patient was counseled regarding the relatively poor predictability in outcome, given the extremely short axial length. On post-operative day 1, the patient’s uncorrected VA was 20/60, which corrected to 20/40 with pinhole, and the patient was asked to come back in 6 weeks to assess vision improvement. At 6 weeks, the OS MRx was recorded as −1.75 −0.75 × 40 degrees, with a best corrected visual acuity (BCVA) of 20/40. To provide further vision correction, the left eye residual refraction was treated with laser *in situ* keratomileusis (LASIK) without complications. His UDVA was 20/25 + 2 in that eye at one month post-operatively with an MRx of plano −0.25 × 16.

**Figure 3:**
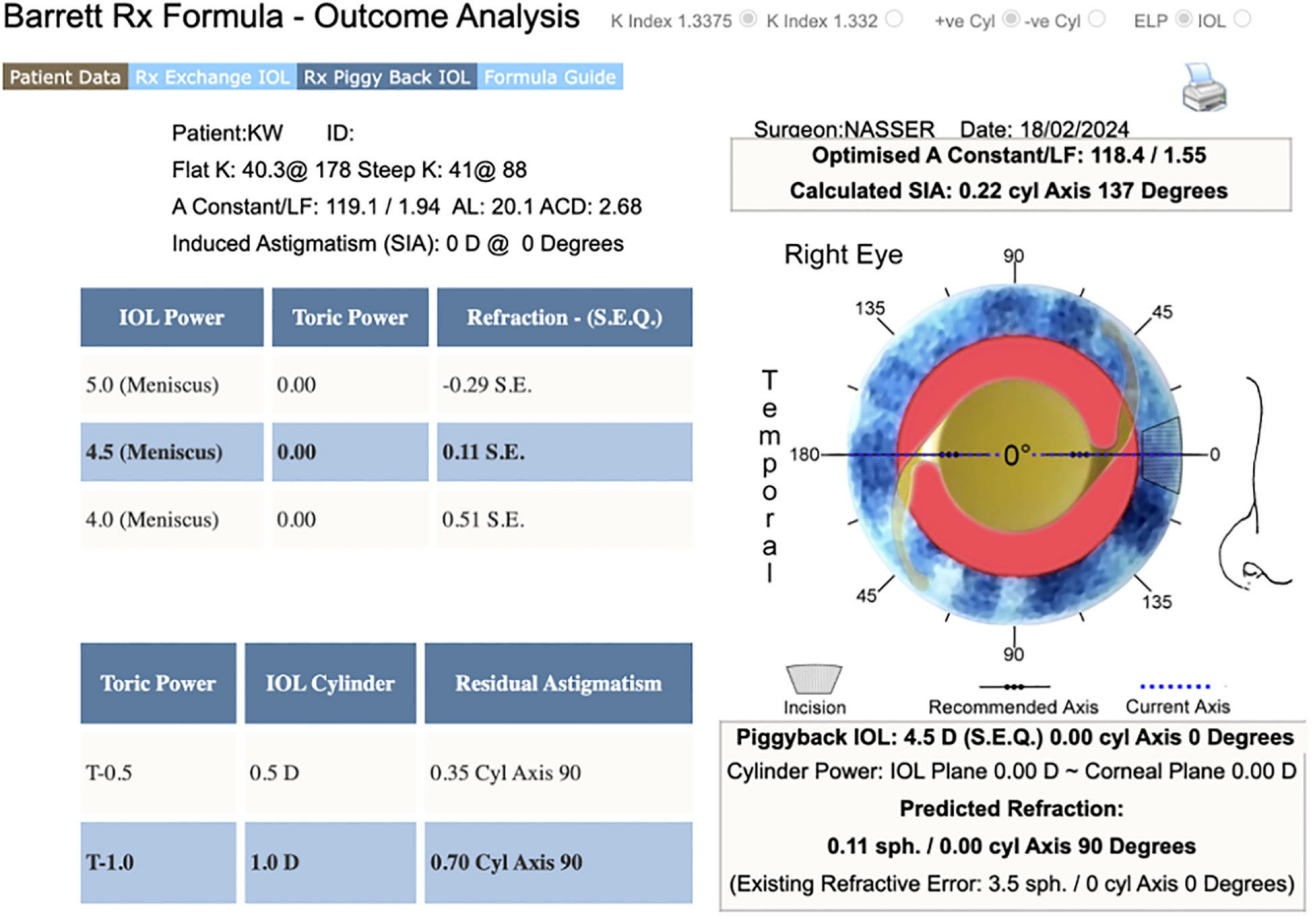
An approximation of a piggyback calculation for the light-adjustable lens using the online Barrett Rx Piggyback IOL calculator.

For the right eye, as seen from **Figure 1**, using a +34.0 D PanOptix lens (Alcon, CCWTT0) was projected to result in approximately +2 to +4 D of residual SEQ. To address this, the average predicted SEQ of formulas was input into the Barrett piggyback calculator (**Figure 3**), which then guided us to target the residual hyperopia with a +5.0 D light-adjustable lens (RxSight, RxLAL). This was also inserted into the capsular bag following the placement of the PanOptix lens (**Video 1**). On post-operative day 1, the patient’s uncorrected VA was 20/25 at distance, 20/20 at intermediate, and J1 at near. At his six-week follow-up, the right eye’s MRx was +1.25 −0.50 × 125 degrees, achieving a BCVA of 20/20. During this visit, the patient underwent ultraviolet light treatment to fine-tune the lens’s power. One week following this treatment, his uncorrected VA was 20/20 at distance, 20/20 at intermediate, and J1 at near with an MRx of +0.50 −0.25 × 32. The patient expressed a high level of satisfaction with the visual outcome in the right eye after a single session of light-adjustable treatment; thus, the lens power was consequently locked in with the routine two sessions.

At the 1-year follow-up appointment, the patient expressed satisfaction with the outcome, noting improved quality of vision and daily comfort compared to pre-operative status. The patient remained clinically stable with maintained visual acuity and intraocular pressure within normal limits. The anterior segment showed no evidence of inflammation or IOL-related complications. The patient demonstrated sustained anatomic and functional stability throughout the observation period. No adverse or unanticipated events were encountered.

## Discussion

Lens replacement surgery is typically undertaken to eliminate the need for glasses. However, residual refractive errors can occur, often due to the effective lens position or measurement inaccuracies. Patients with severe ametropia are particularly at risk for these complications. To correct these residual refractive errors, several surgical options are available. These include exchanging the intraocular lens, employing laser refractive techniques such as LASIK or photorefractive keratectomy (PRK), or the implantation of a piggyback lens.

Our patient, presenting with high hyperopia (+10.00 sphere in OD), was not considered a suitable candidate for laser vision correction. This decision was based on multiple reasons, including insufficient corneal tissue available for ablation, a significant risk of post-operative regression, induction of severe corneal aberrations, and the potential for excessive corneal steepening. Exploring alternative treatments, the option of hyperopic implantable collamer lenses (ICLs) was considered but deemed unsuitable for this patient due to two critical reasons: first, hyperopic ICLs are not available in the United States, and second, the patient’s internal ACD measurements of 2.05 mm in OD and 2.21 mm OS indicated an anterior chamber too shallow for safe ICL implantation. Consequently, lens replacement emerged as the optimal solution for this specific case with the addition of a piggyback lens to address residual refractive errors in OD.

The piggyback technique was first introduced by Gayton and Sanders in 1993 in a case involving cataracts and microphthalmos, in which IOL calculations determined that a power of +46 diopters (D) was needed (6). Traditionally, this method entails the concurrent implantation of both primary and secondary IOLs within the capsular bag. However, an alternative approach involves positioning the primary IOL in the capsular bag and situating the secondary IOL in the ciliary sulcus (7). Employing the piggyback technique offers notable advantages, including a higher safety profile, an easier surgical technique, more reliable power calculation predictions, and the significant benefit of being a reversible procedure (8–10). However, this technique is associated with various potential complications, including post-operative increases in intraocular pressure, pupillary optic capture following mydriasis, iris chafing, pigment dispersion syndrome, secondary pigmentary glaucoma, and interlenticular opacification (ILO) (11–13).

Werner et al. reported that the pathogenesis of ILO is similar to that of posterior capsular opacification, which occurs due to retained/regenerative cortical material (14). In the piggyback technique, the rate of ILO is high when the optics in the bag are both composed of acrylic (15). To address this, placing the secondary IOL in the ciliary sulcus rather than implanting both IOLs in the capsular bag significantly increases the space between the lenses, thereby reducing the likelihood of ILO. Therefore, we took a novel approach to address residual refractive error by piggybacking a light-adjustable lens (RxSight, RxLAL) with a PanOptix lens (Alcon, CCWTT0) in the capsular bag. This decision was driven by the desire to leverage the adjustability of the light-adjustable lens (LAL), offering the distinct benefit of fine-tuning vision correction without the necessity for an additional surgical intervention. This approach is particularly advantageous given the significant variability of outcomes observed in high hyperopic eyes. Furthermore, the LAL features a composition of photo-reactive UV-absorbing silicone, while the PanOptix lens is crafted from hydrophobic acrylic, thereby reducing the risk of ILO. The decision to use a trifocal IOL stems from various studies that have observed improved vision outcomes in patients with hyperopia and presbyopia. Trifocal lens implantation in hyperopic and presbyopic patients resulted in a stable improvement in visual acuity and refraction, along with high patient satisfaction and spectacle independence (16, 17). Even in patients without presbyopia, refractive lens exchange with a trifocal provided improved visual outcomes and high levels of patient satisfaction (18).

In conclusion, this piggyback method was an effective way to yield an extended range of vision for the patient, improving his overall satisfaction and quality of life. The clinical stability of the patient post-refractive lens exchange, including normal intraocular pressures and a lack of inflammation, corroborates the efficacy of this piggyback in providing an improvement in vision without the complications associated with this procedure. While this case is limited in its description of a single patient’s outcome, our approach can be utilized in patients with challenging refractive cases.

## Data Availability

The original contributions presented in the study are included in the article/**Supplementary Material**. Further inquiries can be directed to the corresponding author.

## Supplementary material

Supplementary Video 1 Insertion and intraoperative positioning of primary trifocal IOL and piggyback LAL.
